# Rethinking infrastructure design: evaluating pedestrians and VRUs’ psychophysiological and behavioral responses to different roadway designs

**DOI:** 10.1038/s41598-023-31041-9

**Published:** 2023-03-15

**Authors:** Xiang Guo, Austin Angulo, Arash Tavakoli, Erin Robartes, T. Donna Chen, Arsalan Heydarian

**Affiliations:** 1 Department of Engineering Systems and Environment, University of Virginia, Charlottesville, VA 22904 USA; 2 Department of Civil, Structural and Environmental Engineering, University at Buffalo, State University of New York, Buffalo, NY 14260 USA; 3 Department of Civil and Environmental Engineering, Stanford University, Stanford, CA 94305 USA

**Keywords:** Civil engineering, Information technology

## Abstract

The integration of human-centric approaches has gained more attention recently due to more automated systems being introduced into our built environments (buildings, roads, vehicles, etc.), which requires a correct understanding of how humans perceive such systems and respond to them. This paper introduces an Immersive Virtual Environment-based method to evaluate the infrastructure design with psycho-physiological and behavioral responses from the vulnerable road users, especially for pedestrians. A case study of pedestrian mid-block crossings with three crossing infrastructure designs (painted crosswalk, crosswalk with flashing beacons, and a smartphone app for connected vehicles) are tested. Results from 51 participants indicate there are differences between the subjective and objective measurement. A higher subjective safety rating is reported for the flashing beacon design, while the psychophysiological and behavioral data indicate that the flashing beacon and smartphone app are similar in terms of crossing behaviors, eye tracking measurements, and heart rate. In addition, the smartphone app scenario appears to have a lower stress level as indicated by eye tracking data, although many participants do not have prior experience with it. Suggestions are made for the implementation of new technologies, which can increase public acceptance of new technologies and pedestrian safety in the future.

## Introduction

At its core, infrastructures are in fact an engineering product that have significant impact on people’s day to day lives. However, unlike many other products (e.g., smartphones, computers, etc.), we often overlook the importance of changing the design based on user feedback within the design phase. This is partly due to the fact that such a process can become costly and often not practical in the context of designing infrastructures at the community and city scales^1^. For instance, it is not possible to build different replicas of the same road for testing driver distraction in each alternative design of the road. As a result, many times, design features are chosen by the designer and engineers with minimal feedback (if any) from all end-users (e.g., drivers, bicyclists, pedestrians, scooterstis that will use the road in the future). Over the recent years, due to advancements in technology, designers and decision makers have started to take into account the end user and human factors considerations, especially in the areas of human–building interaction^2^, and human–vehicle interaction^3^. This approach, which is often referred to as a human-centric approach in design, tends to put the user’s needs, comfort levels, and preferences at the center of the design process^4,5^. The integration of human-centric approaches in the infrastructure design has gained more attention recently due to their benefit in different infrastructure systems such as construction safety^6^, accident prevention in traffic safety^7^, energy saving for lighting luminaries^8^, and outdoor comfort in urban spaces^9^.

For the design to become human-centric, it is crucial to measure the factors affecting the human–infrastructure interaction which can be divided into internal and external factors. Internal factors which are mostly related to the user are concerned with users’ preferences, needs, states, and behaviors, while the external factors are related to the outside context that is shaping the users’ environment such as the road environment in a driving condition, and the indoor built environment in a building case^10^. This requires a platform to holistically monitor, model, and analyze the the relationship between the external and internal factors within a human–infrastructure interaction problem. Additionally, it requires methods that can quantitatively provide insights on the perception of the infrastructure from different end-user perspectives (e.g., demographic backgrounds, personality), and simulate different alternative designs within the simulated platform prior to the construction phase. As a new emerging technology, Immersive virtual environments (IVEs) simulators are a promising tool for behavioral studies and to identify how end-users perceive and react to different design alternatives, while holistically monitoring both internal and external factors. Additionally, within IVEs, users have the ability to realistically visualize and interact with the infrastructure before construction and to change the design accordingly. IVE has been applied in many indoor human-building interactions, such as buildings^11–15^ as well as human–transportation interaction problems such as vehicles^16–18^, and cyclists^19^ and other road users. To monitor internal factors, human psycho-physiological metrics such as heart rate, skin temperature, skin conductance, and eye gaze patterns were used in the literature for assessing human state such as stress level, emotion, and cognitive load^10,20–23^. Further, these physiological measures have also shown to be more sensitive than task performance measures to identify task difficulty when using a new technology or exploring a new environment^24–26^. It is easier to implement physiological sensors within IVEs compared to traditional methods such as observational studies or naturalistic studies^27^. Coupling IVEs with psycho-physiological sensing of users allows researchers to measure the internal factors (the end-user perception and response) of alternative infrastructure designs, as well as simulating various external factors, which can objectively prevent faulty design features prior to construction^28^.

This paper aims at describing a system framework to evaluate infrastructure design for the different types of road users (e.g., drivers, pedestrians, cyclists, and construction workers) by leveraging a multimodal IVE system. We conduct a case study within the proposed IVE system to test and assess the users feedback to specific design alternatives for a mid-block crossing infrastructure. For the case study we focus on a transportation infrastructure evaluation for vulnerable road users due to the limited existing research in this area. Designing proper roadway and transportation systems is of high importance as it is highly associated with users well-being, injuries, fatalities, and overall quality of life^29^. However, there is limited attention to how roadway systems need to be designed to be inclusive for all users^30^. The majority of research on roadway design has heavily focused on studies evaluating driver’s behavior, safety, and responses to different design conditions and contextual settings^31^. As a result limited studies have focused on other road users such as pedestrians, bicyclist’s responses to different roadway design and conditions^32^. Among all road users, Vulnerable Road Users (VRUs) such as pedestrians and bicyclists require more attention due to the increasing fatalities in recent years and increasing number of these users^33,34^. Within VRUs, pedestrians are facing more safety challenges on the road, especially during the midwalk crossing as they have less protective equipment and slower speed than the vehicles, scooters, and bicycles^35^. Ensuring the safety of pedestrians is a challenge for researchers, as pedestrian’s decision to cross and the crossing behavior may be affected by many internal factors such as visual/cognitive distraction^36^ and external factors, such as pedestrian infrastructure, roadway design, traffic volumes, vehicle speed, and visibility of the road environment^37,38^. Accidents involving pedestrians are especially common at un-signalized and mid-block crosswalks, where vehicles are less likely to yield to pedestrians^39^.

To increase pedestrian safety at mid-block crossings, different safety treatments have been introduced, such as rapid flashing beacon (RFB)^40^, a vibrotactile wristband^41^, countdown timer^42^, and pedestrian footbridge^43,44^. However, each method mentioned above has its own shortcomings. For example, pedestrians’ response rate to the vibrotactile wristband is low^41^ and the countdown timer could make the pedestrian overestimate their speed, which will result in a higher chance of red light running^42^. The development of connected vehicles and autonomous vehicles has changed the communication environment between pedestrians and vehicles. Recent studies have focused on how to communicate awareness and intent of autonomous vehicles to pedestrians^45^. However, very few studies have pedestrian-centered design, which is how to communicate the pedestrian’s crossing intentions to the vehicles, especially in IVEs^46^. With the limited number of pedestrian study, some researchers have reported the potential of integrating the aformentioned physiological signals in the data collection and analysis. Kitabayashi et al. used heart rate as the biosignal and found out that pedestrians stress in walking are affected by the road congestion^47^. Additionally, pedestrians’ physiological measures were shown significantly correlated with certain urban features such as uneven sidewalks as well as subjective ratings of walkability^48,49^. The physiological data can also be used to quantify the ‘perception-decision-execution’ ability in avoiding danger to a certain level^50^. These studies have been conducted either within naturalistic settings or simulated environments. Within naturalistic settings, researchers are not able to manipulate existing roadway designs or features; meanwhile, within simulated environments, sense of immersion of participants are not realistic, especially on studies conducted on 2D screens. Thus, combining physiological metrics within the IVE, where many design alternatives can be evaluated with high sense of immersion, help us better understand the effect of each pedestrian-centered design on road users.

The provided case study will utilize the framework described to: (1) identify the benefits and limitations of using IVEs for collecting and modeling VRUs’ behaviors and psycho-physiological responses while highlighting how such information could improve the design decision making; (2) evaluate the objective and subjective measures of perceived safety rating across different alternative designs; and (3) evaluate pedestrians’ crossing behavior and psychophysiological responses across different conditions. We will introduce the system framework to collect VRUs’ behavior and physiological response. The system has integrated data collection methods (pedaling/walking performance, eye tracking, heart rate, and video) in virtual reality, and the modularized components makes it applicable to evaluate infrastructure design for all roadway users whether they are pedestrians, bicyclists, scooterists, construction workers, or drivers. Through a case study of pedestrian crossing, 51 pedestrians’ stated preferences, crossing behaviors and physiological responses are collected and analysed with three different mid-block crossing safety treatments—painted crosswalk (as-built), rapid flashing beacons (flashing beacon, Fig. 5), and a connected vehicle phone application (smartphone app, Fig. 6). The goal of this study is not only to identify which design is the best but also to explore the potential benefit of future technology in infrastructure design by implementing virtual reality (VR) method.

This study hypothesises that:

- H1: Null—There are no significant differences in pedestrians’ subjective rating of the perceived safety in the three scenarios scenario. Alternative—at least one scenario’s subjective safety rating will differ from the other two alternatives.
- H2: Null—The pedestrians’ crossing behaviors (wait time, number of stops during the crossing) are similar in all three scenarios. Alternative–The pedestrians’ crossing behaviors will be different in at least one of the two alternative designs.
- H3: Null—Pedestrians in all conditions will have a similar level of cognitive workload, as indicated by mean fixation length, fixation rate, gaze entropy, and mean heart rate. Alternative—Pedestrians would experience significant differences in psychophysiological responses in the flashing beacon and/or smartphone app conditions.

## Results

### Stated preference survey response

In the post-experiment survey, all participants are asked to rate the realism of the IVE with a 5-point Likert scale in several aspects: if the virtual environment feels appropriate to scale (scale), how immersed they felt in the virtual environment experience (immersive), and the extent of consistency of the experiences in the virtual environment with the real-world experiences of crossing a street (consistency). With respect to the scale ratings, an overwhelming majority of participants felt that the virtual environment was to scale (4.1% response ‘3’, 20.4% response ‘4’, 75.5% response ‘5’, mean score = 4.71). Most of them felt immersed in the VR IVE (6.1% response ‘3’, 34.7% response ‘4’, 61.2% response ‘5’, mean score = 4.53). Their experience in the VR IVE was consistent with their real-world experiences when crossing the street (2.0% response ‘1’, 12.2% response ‘3’, 36.7% response ‘4’, 49.0% response ‘5’, mean score = 4.31). Except for one participant responsed with ‘1’ in consistency rating, most participants reported a high realism of the simulator.

On average, participants have a higher safety rating in flashing beacon scenario (4.56 out of 5 scale), followed by the smartphone app (3.6), and the as-built environment (3.0). The differences between the safety rating are all significant at a 95% confidence level ($\alpha = 0.05$).

**Figure 1:**
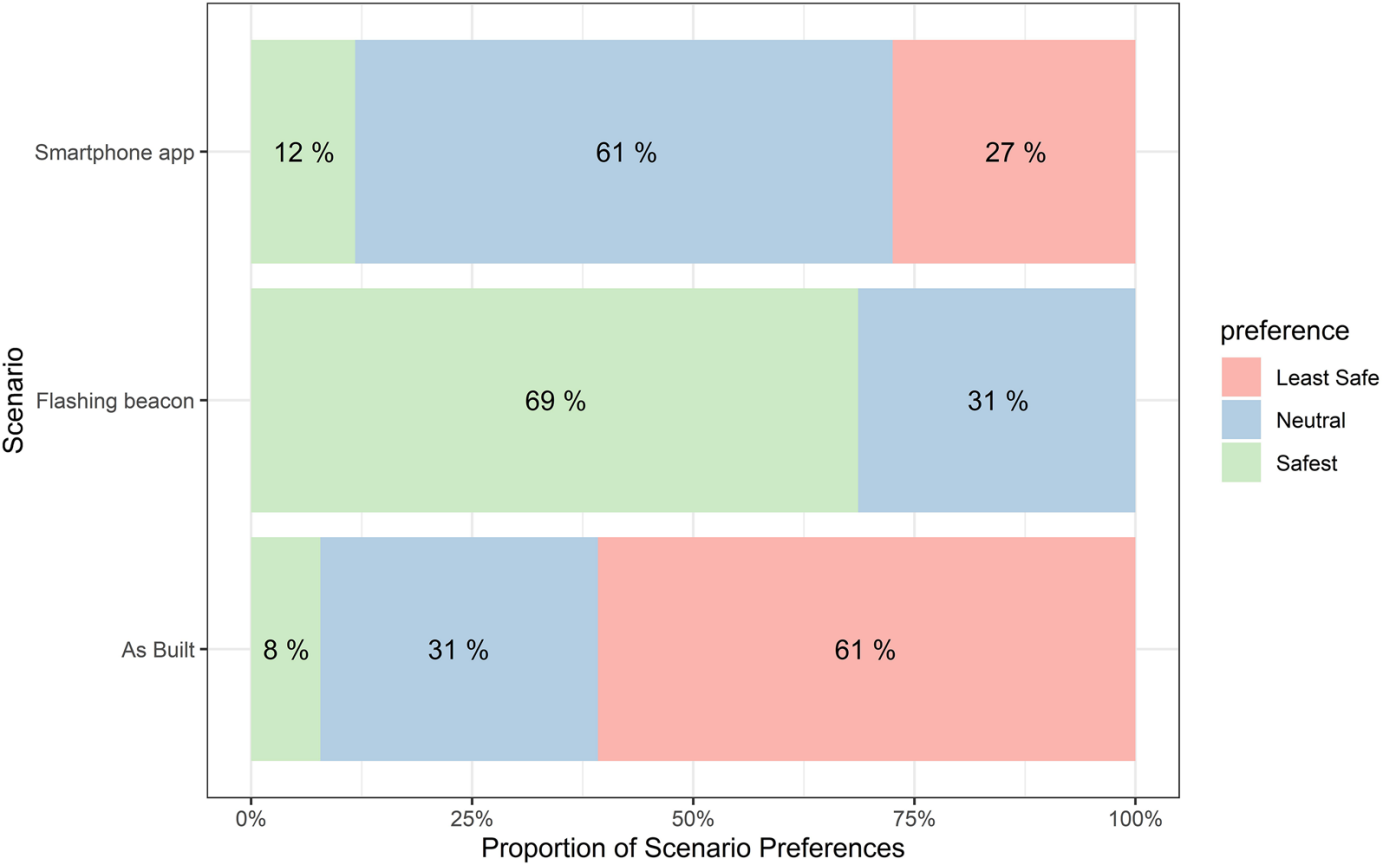
Safety preference for different scenarios from survey response.

Additionally, once asked to rank the three environments based on their perceived safety measures from safest to least safe, participants’ responses supported the previous metric, with flashing beacon ranked as the safest and the as built environment as the least safe condition (Fig. 1). 69% of the participants rate the flashing beacon scenarios as the safest scenario of the three options, none of them rate it as the least safe scenario. For smartphone app scenario, 12% rate it as the safest, 27% participants rate it as the least safe one. For the as-built scenario, only 8% rate it as the safest and 61% of the participants rate it as the least safe scenario.

### Crossing behavior

#### Crossing time

**Figure 2:**
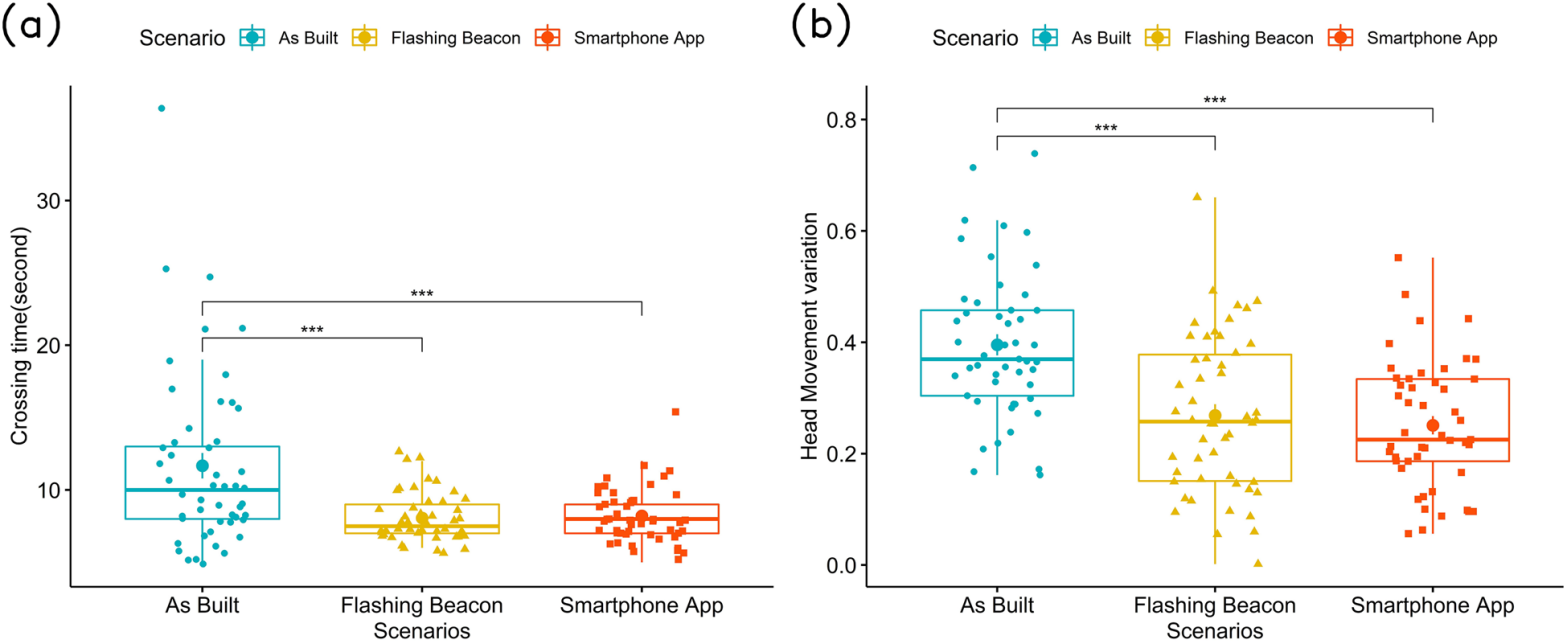
(**a**) Average crossing time of different scenarios; (**b**) head movement variation of different scenarios.

For the crossing time, as shown on Fig. 2a, participants had a significantly lower crossing time in the flashing beacon ($\beta = -3.604, SE = 0.717, p = 0.0026$) and smartphone app cases ($\beta = -3.417, SE = 0.720, p = 0.00013$) as compared to the as-built environment. No significant differences are found between the flashing beacon and smartphone scenarios.

#### Wait time before crossing

The wait time before crossing for as-built, flashing beacon and smartphone app scenarios are 20.34 s, 22.20 s, and 21.47 s respectively. A marginal significant difference between as-built and smartphone app scenarios is found ($\beta = 2.284, SE = 1.177, p = 0.0548$).

#### Wait time after crossing decision

The wait time after crossing decision for smartphone app scenario (mean = 4.23 s, sd = 2.92 s) is lower than flashing beacon scenario (mean = 5.20 s, sd = 3.35 s), but the difference is not significant.

#### Head movement

The result shows a significant difference between the as-built and the two other scenarios with both p values less than 0.001. Specifically, for the flashing beacon scenario,$\beta = -0.118, SE = 0.0284, p = 0.0000657$, and for the smartphone app scenario, $\beta = -0.144, SE = 0.0241, p = 4.50e-08$. However, we did not find a difference between the flashing beacon and smartphone app scenario. As shown on Fig. 2b, participants had a higher variation of head movement direction in the as-built environment as compared to the other two scenarios. It is also shown from the results that low prior VR experience contributes to higher head movement variation ($\beta = 0.069, SE = 0.027, p = 0.0141$).

#### Stop during crossing

We manually annotated the experiment videos to determine if the pedestrians have stopped in the middle of their crossing. Two participants’ (participants 42 and 46) data are excluded due to failure in video recording. Therefore, 49 participants’ stop behaviors are recorded. As shown in Table 1, Pedestrians in the as-built scenario stop significantly more in the middle of the corss walk compared to the other two scenarios. Interestingly, the flashing beacon and smartphone app scenarios has exact the same number of stops across the participants. In both scenarios, there are 10 participants who stop in the middle of crossing to wait for the vehicle’s response although they are told that the vehicles will stop for them after they send their request by pushing the buttons.

**Table 1:** Number of pedestrians’ stops during crossing.

Scenarios	No stop cases	Stop cases
As-built	19	30
Flashing beacon	39	10
Smartphone app	39	10

### Eye tracking

For the eye tracking data, five participants’ data are excluded due to hardware failure during data collection. The eye tracking results in this section are based on 46 participants’ data.

#### Fixation

**Figure 3:**
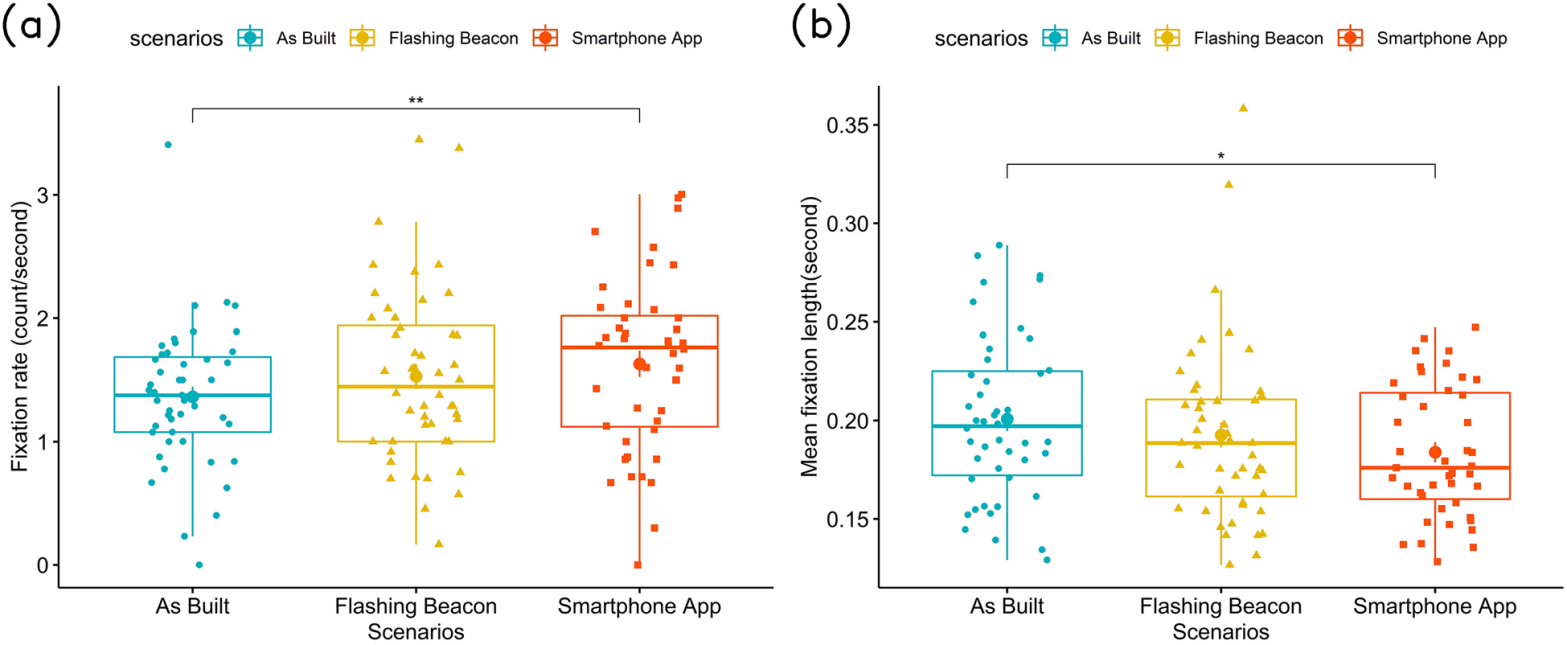
Fixation measurement during crossing of three scenarios, (**a**) fixation rate, (**b**) mean fixation duration.

Participants in the smartphone app scenario had a significantly higher fixation rate as compared to the as-built environment ($\beta = 0.235, SE = 0.111, p = 0.0369$). We did not find any significant differences between the other scenarios, as shown in Fig. 3a. Furthermore, male participants’ fixation rate are significantly lower than females ($\beta = -0.296, SE = 0.145, p = 0.0475$). Participants with a low level of familiarization to VR devices have a lower fixation rate ($\beta = -0.531, SE = 0.050, p = 0.000894$).

For mean fixation duration, there is a significant difference between the as-built and the smartphone app scenarios ($\beta = -0.0179, SE = 0.00787, p = 0.0259$). As shown on Fig. 3b, participants had a lower mean fixation duration in the smartphone app scenario with an average of 0.184 sas compared to the as-built environment with an average of 0.201 s, in between is the mean fixation duration of flashing beacon scenario (0.193 s).

#### Gaze entropy

**Figure 4:**
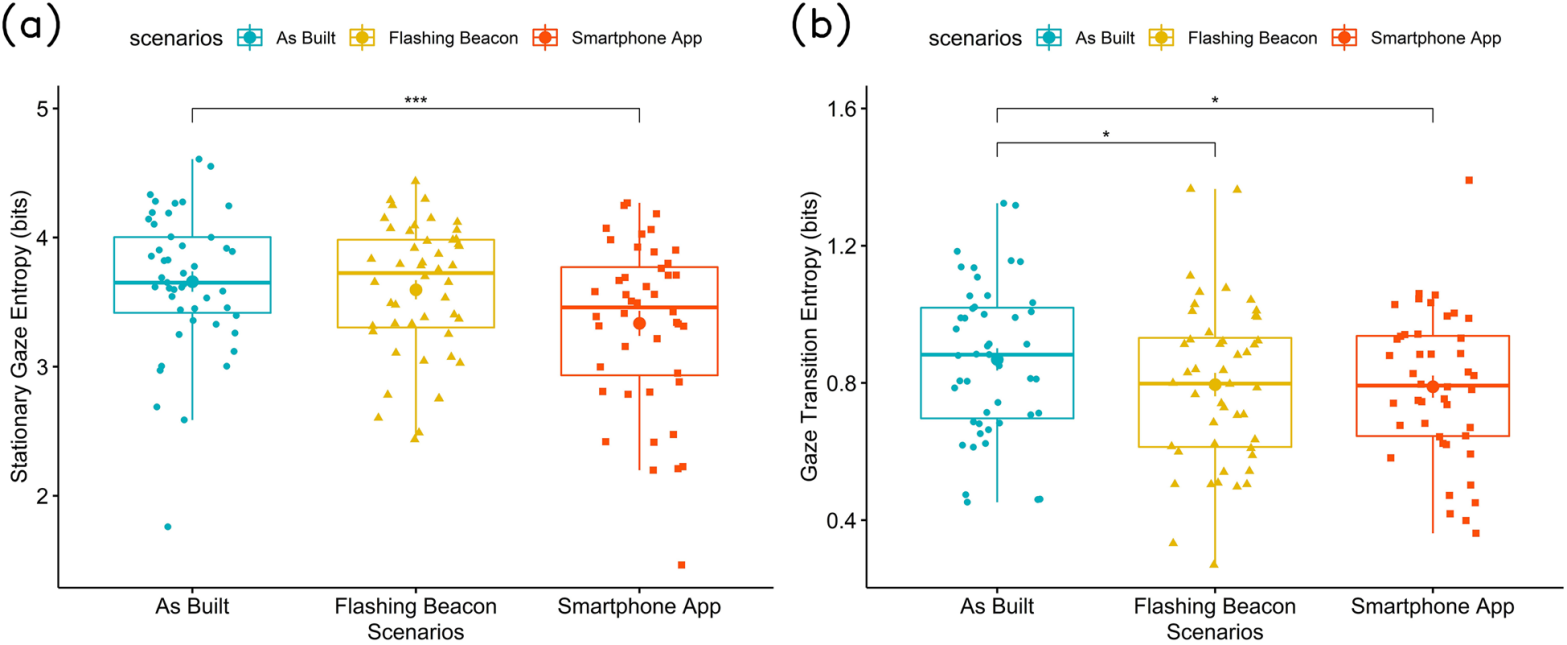
Gaze entropy during crossing of three scenarios, (**a**) stationary gaze entropy, (**b**) gaze transition entropy.

There are two types of gaze entropy measures: stationary gaze entropy (SGE) and gaze transition entropy (GTE). The results for the SGE shows that participants had a significantly lower SGE in the smartphone app as compared to the as-built environment, as shown in Fig. 4a. ($\beta = -0.343166, SE = 0.092471, p = 0.00036$). Older pedestrians have a overall lower SGE than younger pedestrians ($\beta = -0.011, SE = 0.005, p = 0.0439$). The results of GTE shows that the GTE is significantly lower in the both flashing beacon ($\beta = -0.0764, SE = 0.0403, p = 0.0444$) and smartphone app scenarios ($\beta = -0.0830, SE = 0.0411, p = 0.0435$) as compared to the as-built environment. No significant results are found between the flashing beacon and smartphone app scenario, as shown in Fig. 4b.

### Heart rate

The heart rate result indicates that there is no significant differences between the three scenarios a 95% confidence level. Marginal difference for the mean heart rate during crossing is found between the smartphone app scenario and the as-built scenario ($\beta = -1.909, SE = 1.109, p = 0.0886$). The mean HR (beat per minute) of the as-built, flashing beacon and smartphone app are 86.40, 86.29 and 84.63, respectively.

## Discussion

Overall, from stated preference results, both the flashing beacon and smartphone app scenarios are perceived to be safer than the as-built scenario, and the participants show a higher preference on the flashing beacon scenario based on both the subjective and objective ratings. The majority of the participants (69%) choose the flashing beacon as the safest scenario, which could imply their trust in this technology (as well as their familiarity with this technology as it exists on some roads).

Interestingly, the results from crossing behavior and physiological responses are slightly different from stated preference. For average crossing time, both the flashing beacon and smartphone app scenarios have a lower average crossing time compared to the as built scenario; additionally, there is no significant differences between the flashing beacon and smartphone app crossing time. The pedestrians have a lower wait time before crossing, but spend more time during the crossing, this is aligned with an observational study conducted by^35^ at mid-block crosswalks in which pedestrians who waited for little or no at the curbside generally lost time during the crossing.

It is important to also note that some participants indicated that they were not sure about the smartphone app performance, so they chose to wait until the vehicle came to a complete stop for them. Our records showed that seven participants stated that they were not sure about what will happen after they pressed the button on the smartphone, more feedback in the smartphone app scenario is desired, as indicated by some comments, “*It will great to know if the nearby vehicles received my request when using the App, maybe a feedback on your phone, like message saying received by coming vehicles.*” (P6) and “*I’m little concerned about using the phone app to inform the drivers, because I have no experience on that.*” (P21).

However, when checking the waiting time after crossing decision, the smartphone app scenario actually has a lower average waiting time after crossing decision (4.23 s) than flashing beacon scenario (5.20 s), although the difference is not significant. This may be explained by different reactions required by the two interactions (press the button on the phone vs. physically reach out to the button), or the gap acceptance difference in the two scenarios.

With respect to head movement, the larger head movement variation in the as-built scenario indicates that participants are more hesitant during crossing, while no significant differences are found between the two alternative designs. Furthermore, visual inspection of the videos also qualitatively verifies the fact that the proportion of stop behaviors during crossing are the same for the two alternative designs, and both are lower than the as-built scenario.

For eye tracking data, the difference in fixation rate and mean fixation duration between the as-built and smartphone app scenario shows pedestrians’ different visual scanning strategies. The longer fixation duration in the as-built scenario means that pedestrians spent a long time on searching the environment and potential hazards. As reported by previous studies, longer fixation duration and lower fixation rate is related to higher cognitive load^15^. An earlier pedestrian eye tracking study also found that ‘safe’ pedestrians have a lower mean fixation duration than ‘rogue’ pedestrians after they get used to the environment^51^. Lower SGE and GTE are observed in the smartphone app, as far as we know, there is no existing studies about the pedestrian gaze entropy. In flight situations, low gaze entropy is usually accompanied by high situation awareness, for different tasks, the gaze entropy of the group that succeeded in the task was low^52^. Therefore, our results may indicate that the smartphone app scenario may have a lower cognitive workload for the pedestrian to cross.

Due to the relatively low HR data frequency, only a limited number of HR data points are utilized for the mean HR comparison. Marginal significantly lower mean HR is found for smartphone app scenario in this study, which may reveal a lower stress level in the smartphone app scenario as compared to the other two scenarios. As mentioned before, previous studies show that lower HR values are generally associated to calmer, less-stressful states^21^. However, we note that this finding needs to be validated in the future study with more professional HR data collection devices. In addition, the fidelity of the IVE system can be another reason that contributes to the significance of HR results, although our framework features with a simulation from a real-world environment, a head-mounted display for visualization, the real-time agency of movement, and environmental sound, more steps can be taken to further improve the fidelity such as the simulation of other pedestrians, weather conditions, and haptic feedback.

The qualitative feedback collected from participants may also help to find the reasons behind the differences in subjective ratings and objective responses. A couple of participants stated that they were not sure about what would happen in the smartphone app scenario after pressing the button on the screen although instructions are given before the experiment. This may be the reason why more participants prefer the flashing beacon scenario. However, the crossing behavior data shows that the waiting time after crossing decision for the smartphone app is not significantly different from flashing beacon. For other crossing behavior variables, we also do not find significant differences between the flashing beacon and smartphone app scenario. In addition, for physiological responses, the smartphone app scenario seems to have a slightly better overall performance with a shorter fixation duration, higher fixation rate and lower HR, which is related to lower cognitive load. Given the fact that there is still much room for improvement in the smartphone app scenario, a better physiological performance can be expected if such limitations are addressed.

Our results further emphasize the importance of objective measurement for the evaluation of infrastructure designs as the users’ subjective answers may not reflect their actual behaviors. The difference in subjective ratings and objective responses also highlights that public education is an important step of new technology implementation. In our study, although the smartphone app scenario shows a good overall performance, participants do not have a high safety ratings on it because they do not have any related experience with the new technology. IVE-based simulation offers a risk-free and low-cost platform for the public to get familiar with new technologies, which will help to increase the acceptance of these new technologies which are currently not familiar to them.

## Limitations and future work

The eye tracking section of our study only focuses on the overall information of fixations (fixations rate and mean fixation duration) and the general distribution of the fixations (gaze entropy), it makes more sense to extract contextual information about fixations. By defining Area of Interests (AOIs) such as the button on the flashing beacon, smartphone, crosswalk path or other vehicles, it would help to gain a better understanding of what the pedestrians are looking at. The visual attention allocation of pedestrians will provide more information about distraction state^53^. In our future study, in-depth analysis of eye tracking data by integrating the AOIs information will be performed to explore pedestrians’ visual attention allocation on key AOIs, such as the flashing beacon button, the smartphone and the vehicles.

Another limitation of our study was the low frequency of HR data. Due to collecting HR using off-the-shelf smartwatches we did not have access to higher frequency physiological sensing. Future work should consider adding other physiological sensing modalities such as skin temperature and skin conductance to enhance the physiological sensing module and inference. However, it should also be considered that more devices might degrade the feeling of realism of the study. More advanced devices that can collect multiple physiological sensors simultaneously can be integrated into studies as such to keep the realism while recording a higher number of modalities of data.

Other limitations, as also mentioned by the participants were to include (1) realistic vehicle actions, (2) feedback from the smartphone app, and (3) traffic simulation. Currently, we are improving the logic of the vehicle by refactoring the vehicle speed controller so the response will be more realistic. More ways of interactions and the feedback are being developed such as audio warning, tactile feedback from the controller, vehicle’s flashing light, projections on the crosswalk, and so on. The various ways of interactions will be evaluated by users’ stated preferences and objective responses as well. Moreover, based on our framework^27^, it is possible to include multiple agents in the IVE, so other road users such as bicyclists and drivers can be studied together with pedestrians within the IVE. We are developing a multi-agent simulator for different road users (more pedestrians, cyclists, work zone workers, and drivers) based on the current system framework, aiming to study the pedestrian platoons simultaneously in the same VR environment. More results are expected in our follow-up papers.

## Conclusion

This paper presents the evaluation of three pedestrian crossing infrastructure designs (the as-built painted crosswalks, the flashing beacon and a connected vehicle phone application) in an IVE-based experiment. With the system framework, the stated preferences, crossing behavior and physiological responses are collected from 51 participants. Several advantages can be identified from this study over an observational study: First, it is possible to collect physiological responses, such as eye tracking and heart rate. Second, this type of study can guarantee experimental control over other factors that may affect the response, such as weather conditions, traffic volumes, and other infrastructure conditions. Third, the designs that are currently unavailable in the real world can be evaluated in the IVE, such as the connected vehicle technology. Lastly, the IVE-based study offers a risk-free and low-cost platform, especially for the underrepresented road users, such as females, disabled and elderly people. The results indicate that the two alternative designs have a higher safety ratings than the as-built scenario, and the flashing beacon scenario is rated as the safest. Pedestrians in the as-built scenario have a lower waiting time but spend/lost more time during crossing by stopping in the middle of the crosswalk to wait for the vehicle, in addition, a larger head movement variation is observed in the as-built scenario. The crossing behavior in the flashing beacon and smartphone app scenario is similar. For the eye tracking data, pedestrians had a shorter fixation duration, larger fixation rate, smaller stationary gaze entropy and smaller gaze transition entropy in the smartphone app than the as-built scenario, which may be resulted from a lower cognitive workload. The difference between the flashing beacon and as-built scenario is not as significant as the smartphone app. A marginal significant lower mean heart rate is found in the smartphone app scenario. Overall, both the flashing beacon and smartphone app have a better physiological performance than the as-built scenario, but the smartphone app scenario appears to have a slightly better physiological outcome. Qualitative feedback is collected from the participants to explore the reasons for the differences between stated preferences and objective measurements, discussions, and suggestions are made. In conclusion, public education is required before the implementation of new technologies such as connected vehicles, which can help to increase users’ acceptance and safety.

## Methods

The study is reviewed and approved by the Institutional Review Board for the Social and Behavioral Sciences from University of Virginia (IRB-2148). All experiments were performed in accordance with relevant named guidelines and regulations. Informed consent was obtained from all participants and/or their legal guardians.

### Study design

This research designs a within-subject experiment to study pedestrians’ stated preferences, crossing behavior, and physiological responses to three different mid-walk crossing designs in an immersive virtual environment with a random order: painted crosswalk (as-built), rapid flashing beacons (flashing beacon), and a connected vehicle smartphone application (smartphone app). The selected location for this study is the intersection of Water St and 1st Street South in Charlottesville, Virginia. This place has been identified as a hotspot for pedestrian-vehicle accidents in the Virginia Department of Transportation’s Pedestrian Safety Action Plan^54^. The intersection of Water Street and 1st Street South is chosen as the study site. The north side of the intersection is a dead-end road (utilized only for deliveries). The south side of the road is a one-way street, which vehicles cannot turn onto from Water Street.

At the beginning of the experiment, each participant is asked to sign the consent form approved by the IRB office and put on two smartwatches on both wrists, before completing the pre-experiment survey. After finishing the pre-experiment survey, instructions are given on how to use the VR headset, controllers, and pedestrian simulator, as well as how the three scenarios are designed and how to interact with the infrastructures in the VR. After the IVE system setup, the participant is placed into a familiarization scenario without any vehicle traffic to become familiar with interacting with the IVE. In this environment, the participant is free to walk around in the given area until the participant feels comfortable. Then the participant will experience the three scenarios in random order. In each scenario, pedestrians will be placed into the beginning location, facing the crosswalk heading southbound along 1st Street, crossing Water Street from the north side of the road. The independent variables are the crossing infrastructure designs and demographic information (i.e., age, gender). The dependent variables are stated preferences of the three scenarios, crossing behavior (crossing time, waiting time before crossing, waiting time after crossing decision, stop or not during crossing, and head movement variation) and physiological responses (i.e., eye tracking and HR features) during crossing.

### Virtual reality system setup

A one-to-one road environment is built in the Unity software with SteamVR platform. HTC Vive Pro Eye headsets with the controllers are utilized for any interactions in the IVE. More detailed information of the IVE setup is available in our previous studies^19,27,55^. Vehicle traffic within the IVEs is generated from empirical gap acceptance data observed at the real-world location. The gaps between vehicles are generated to fit the empirical distribution of accepted gap sizes^55^. These gaps are randomized before each scenario so each participant’s exposure to any gap is randomized. All the vehicles has a speed of 25 mph, followed the speed limit. Vehicle type is also randomized from the four vehicle models used in the IVE.

#### As-built scenario

The as-built environment is built to model the existing painted crosswalk along the Water Street corridor to serve as the base case against the other two alternative designs. In the IVE, the pedestrian’s task is to crossing the street when they feel safe to do so after the first vehicle passes the crosswalk. The vehicle will stop right before the crosswalk to wait for the pedestrian to cross if a conflict is expected to happen.

#### Flashing beacon scenario

**Figure 5:**
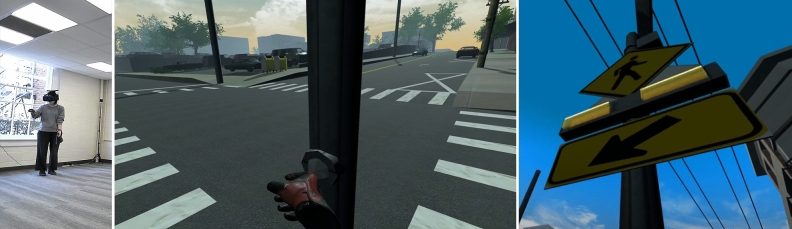
A pedestrian is using a flashing beacon for crossing (left), the pedestrian point of view in IVE (middle), the flashing beacon in IVE (right). A sample video is available in the following link https://youtu.be/hz64mFP83LA.

In the flashing beacon scenario, the pedestrian is allowed to cross the road whenever they feel appropriate. Pedestrians are able to interact with the flashing beacon by pressing the button located on the sign pole to initiate the flashers on the beacon. Figure 5 shows how a pedestrian interacts with the RFB while in VR prior to crossing, as well as an image of the RFB in VR when used.

#### Smartphone application

**Figure 6:**
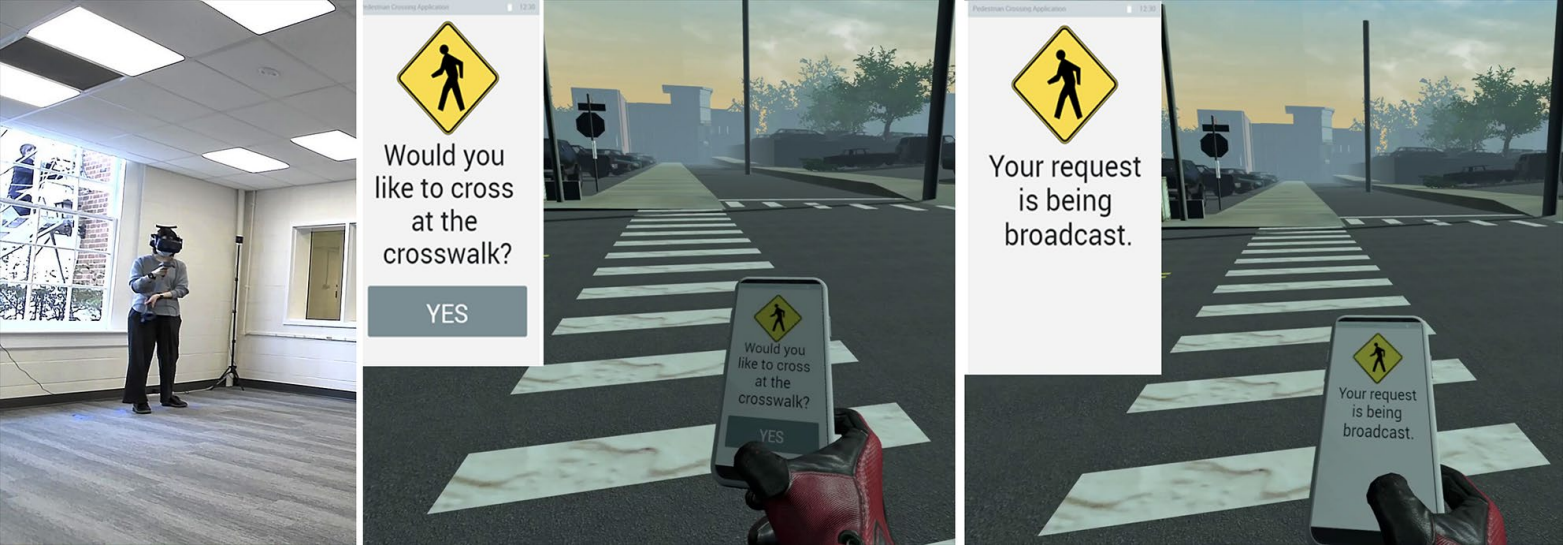
A pedestrian is using smartphone app (left), pedestrian’s point of view in IVE (middle), smartphone app user interface before and after pressing (right), a sample video is available in the following link https://youtu.be/Q_LUoIZuPKs.

In the smartphone app scenario, pedestrians will have a cellphone (a controller in their right hand in real life) in their right hand once they are placed in the IVE. As shown in Fig. 6, there are two interfaces that will show up on the phone during testing. The first interface of the mobile phone application (initial state) asks the pedestrian if they wish to cross the crosswalk. Should the pedestrian answer “Yes” and press the button on the controller’s central pad, a new interface will pop up indicating “Your request is being broadcast”. Once the system detects the pressed button, the program will send the request to the next approaching vehicle, and then it will brake and stop in front of the crosswalk to yield to the pedestrian, all the follow-up vehicles will stop as well. The pedestrian is then free to cross the crosswalk and vehicles will yield before the crosswalk for the pedestrian.

### Data collection

The data collection method of this study follows the framework of our previous study^27^, different types of behavioral and physiological data are collected: stated preferences from pre and post experiment survey, crossing behavior data from Unity, eye tracking data from Tobii Pro Eye headset, heart rate data from smartwatches.

#### Survey response

In addition to demographic information, in the pre-experiment survey, the participants are also asked to provide their familiarity with VR devices. After the experiment, the participants are asked for their safety ratings and preferences over the three scenarios. For each scenario, they will be asked to provide their answer with a Likert Scale 1–5 to the question “How safe do you feel in the scenario”, where 1 indicates “not safe at all” and 5 indicates “very safe”. Furthermore, they are asked to rank the safest to the least safe scenario from the three environments.

#### Crossing behavior

Five response variables are recorded to represent the pedestrians’ crossing behavior: crossing time, waiting time before crossing, waiting time after crossing decision, stop or not during crossing, and head movement variation. The crossing time is defined as the time interval from the moment when the pedestrian start crossing to the moment when the pedestrian reaches the other side of the crosswalk. Waiting time before crossing is defined as the time between the start of the experiment and the moment when the pedestrian start crossing. Waiting time after crossing decision are defined as the waiting time after pedestrian’s decision to cross the street (after pressing the button either on flashing beacon or smartphone to start crossing), which is only accessible in the flashing beacon and smartphone app scenarios. Stop or not during crossing is a binary response about whether the pedestrian has a obvious stop to wait for the vehicle’s behavior during crossing. The head movement is defined as the variations in the 3-D head movement direction in the unit vector.

#### Fixation

Fixation is defined as the moments when eyes stop scanning about the scene and hold the central foveal vision in certain places to look for detailed information of the target object. Similar to previous studies^28,56^, We define a fixation with 25 ms minimum duration and 100 pixel maximum dispersion thresholds to extract the fixation information from the original eye tracking data and videos. Two measurements of fixation are calculated: (1) the mean fixation duration is defined as the average length of all fixation events during the crossing; and (2) the fixation rate is defined as the number of fixations per second during the crossing.

#### Gaze entropy

there are two types of gaze entropy measures: stationary gaze entropy (SGE) and gaze transition entropy (GTE). SGE provides a measure of overall predictability for fixation locations, which indicates the level of gaze dispersion during a given viewing period. The SGE is calculated using Eq. (1):

1 $$\begin{aligned} H(x) = -\sum _{i=1}^{n}(p_i)log_2(p_i) \end{aligned}$$

*H*(*x*) is the value of SGE for a sequence of data *x* with length *n*, *i* is the index for each individual state, $p_i$ is the proportion of each state within *x*. To calculate the SGE, the visual field is divided into spatial bins of discrete state spaces to generate probability distributions. Specifically, the coordinates are divided into spatial bins of $100 \times 100$ pixel. *i* to *n* is defined as all the gaze data during crossing.

GTE is retrieved by applying the conditional entropy equation to first order Markov transitions of fixations with Eq. (2):

2 $$\begin{aligned} H_{c}(x) = -\sum _{i=1}^{n}(p_i) \sum _{i=1}^{n}p(i,j) log_2 p(i,j) \end{aligned}$$

Here $H_{c}(x)$ is the value of GTE, and *p*(*i*, *j*) is the probability of transitioning from state i to state j. The other variables have the same definitions as in the SGE equation (1). More details of calculating SGE and GTE can be found in^27,28^.

#### Heart rate

An Android smartwatch with the “SWEAR” app^57^ records the HR data with a frequency of 1 Hz. The watch is connected to a smartphone via Bluetooth, and the time is synchronized with the experiment computer before each experiment. All data from the smartwatch is temporally stored on the local device and then uploaded to Amazon S3 cloud storage to download for further analysis.

### Participants

51 participants were recruited for the experiment. Most of the participants are local residents, university students, and faculty members who are familiar with the study corridor. All participants are 18 or older and without color blindness. Two participants’ data are removed due to the malfunction in the data collection. For the remaining 49 participants (22 female and 27 male), the mean age is 33.92 with a standard deviation of 12.95 (1 participant did not reveal his/her age information).

### Statistical modeling

A Linear Mixed Effects Model (LMM) was chosen to model the different response variables between independent variables across participants^58^. The LMM framework is chosen specifically for their ability in addressing random and main effects simultaneously within the same modeling scheme^58^. This type of modeling allows us to investigate the effect of each independent variable by considering that each participant might have different baselines for their psychophysiological responses.

An LMM is defined as the following:

3 $$\begin{aligned} y = X\beta + bz +\varepsilon \end{aligned}$$

In Eq. (3), *y* is the dependent variable in our problem, *X* is the matrix of predictors, $\beta$ is the vector of fixed-effect regression coefficients, *b* is the matrix of random effects, *z* is the coefficients associated to each random effect, and $\epsilon$ is the unexplained error terms. The *b* and $\epsilon$ matrices are defined as:

4 $$\begin{aligned} b_{ij}\sim & {} N(0,\psi _k^{2}),Cov(b_k,b_{k'}) \end{aligned}$$

5 $$\begin{aligned} \varepsilon _{ij}\sim & {} N(0,\sigma ^{2}\lambda _{ijj}),Cov(\varepsilon _{ij},\varepsilon _{ij'}) \end{aligned}$$

In our modeling, we applied LMM using the *lme4* package in R programming language^59^.

The independent variables are the demographic information (age, gender), prior experience with VR devices (categorized as high/low by if they have used any VR devices before), and the three different pedestrian crossing designs. The dependent variables are all the behavioral responses, including crossing behaviors (crossing time, wait time before crossing, wait time after crossing, head movement and stop during crossing), eye tracking (fixation and gaze entropy), and heart rate. This analysis was performed in R programming language^60^ using the LME4 package^59^. All statistical analyses were performed at a 95% confidence level ($\alpha = 0.05$).

## Data Availability

The datasets analysed during the current study are available in the Open Science Framework repository—“Supplemental materials for paper: rethinking infrastructure design: evaluating vulnerable road users psychophysiological and behavioral responses to different design alternatives” repository with the link of https://osf.io/8w29f/.
